# Supplementary material for: Double-cuff versus single-cuff bronchial blockers for video-assisted thoracoscopic lobectomy: A randomized controlled trial
Source: PLoS One. 2026 May 21;21(5):e0350007. doi: 10.1371/journal.pone.0350007 (PMC13193552; doi:10.1371/journal.pone.0350007)
Supplement: S1 File — (DOCX) [file pone.0350007.s001.docx]

**Research Protocol**

(Version: 1.0, Version Date: 2024-6-1)

**Project Name**: **Comparison of the application of double cuffs bronchial blocker and single cuff bronchial blocker in thoracoscopic lobectomy**

**Project Name**: Comparison of the Application of Double-cuff bronchial blocker and Single-cuff bronchial blocker in Thoracoscopic Lobectomy

**Sponsor:** Nanpi People's Hospital

**Department**: Department of Anesthesiology and Surgery

**Principal Investigator**: Guoliang Tan

## Investigator's Declaration and Protocol Signature Page

As the principal investigator of this research project, I will abide by the Ethical Review Measures for Biomedical Research Involving Human Subjects (2016) of the Ministry of Health, the Helsinki Declaration (2013) of the World Medical Association, the International Ethical Guidelines for Biomedical Research Involving Human Subjects (2002) of the Council for International Organizations of Medical Sciences, and the ethical principles of Good Clinical Practice (GCP). Under the guidance of GCP, I will conduct the research using the protocol approved by the ethics committee to ensure the scientific nature of the research and protect the health and rights of the subjects.

**Name**: Guoliang Tang

**Signature**: ________________

**Date**: ________________

## Protocol Abstract

| Protocol Title | Comparison of the application of double cuffs bronchial blocker and single cuff bronchial blocker in thoracoscopic lobectomy |
| --- | --- |
| Version Number/Version Date | Version 1.0/2024-6-1 |
| Sponsor | Nanpi County People's Hospital |
| Principal Investigator | Tang Guoliang |
| Study type | intervention study |
| Objectives of Study | This study was to observe and compare the safety, effectiveness and prognosis of the application of double cuff bronchial blocker and single cuff bronchial blocker in thoracoscopic lobectomy, so as to provide the basis for the selection of appropriate airway management tools during clinical one lung ventilation.。 |
| Sample size | 80 cases, including 40 cases in the double-cuff bronchial blocker group and 40 cases in the single-cuff bronchial blocker group |
| Research Subjects | It is planned to select 80 patients who will undergo thoracoscopic lung tumor resection with bronchial blocker technology in Nanpi People's Hospital |
| Research Method | 80 patients who were scheduled to undergo thoracoscopic lung tumor resection with bronchial blocker technology in our hospital were randomly divided into the double-cuff bronchial blocker group (n = 40) and the single-cuff bronchial blocker group (n = 40). After the patients entered the room, routine monitoring was carried out. After anesthesia induction and endotracheal intubation, the double-cuff bronchial blocker was inserted into the tracheal tube in the double-cuff bronchial blocker group; the single-cuff bronchial blocker was inserted into the tracheal tube in the single-cuff bronchial blocker group. The blood gas results, lung isolation effect, and patient prognosis during one-lung ventilation were observed in the two groups. |
| Inclusion Criteria | (1) 80 patients who were scheduled to undergo thoracoscopic lobectomy in our hospital were selected; (2) American Society of Anesthesiologists (ASA) physical status classification was grade I - II; (3) Age 18 - 65 years old, body mass index (BMI) 18.5 - 25 kg/m²; (4) No obvious abnormality in lung function, forced expiratory volume in one second/forced vital capacity (FEV1/FVC%) > 70%, no restrictive or obstructive ventilatory dysfunction; (5) No obvious abnormality in cardiac function, no cardiovascular disease, ejection fraction (EF) > 50%; (6) No anemia or other hematological diseases before surgery and no history of radiotherapy or chemotherapy; (7) Agree to participate in this study and sign the informed consent form. |
| Exclusion Criteria | (1) The patient or their family members refuse to participate in the trial; (2) Moderate or severe abnormality in cardiopulmonary function; (3) History of bronchial asthma and airway hyperresponsiveness; (4) Patients with pulmonary infection, bronchopleural fistula, emphysema, or bullae; (5) The length of the right main bronchus is less than 0.5 cm; (6) Patients with contraindications for using the blocker, such as tumors in the airway. |
| Trial End Criteria | All the required experimental design data have been collected. |
| Withdrawal/Exclusion Criteria | All the required experimental design data have been collected; intraoperative hypoxemia (SPO2 ≤ 92%) occurs in the patient; intraoperative massive hemorrhage, allergic reaction, arrhythmia, and other unexpected situations occu |
| Research Progress Plan | Complete ethical review and register in the Chinese Clinical Trial Registry from June to August 2024. Complete case collection from September to December 2024. Complete experimental statistics and paper writing from January to March 2025. Complete paper publication from April to December 2025 |
| Statistical Analysis Method | Statistical software SPSS21.0 version was used for analysis. For measurement data that meet the comparison of means of single-factor multiple samples and pass the normality test and variance homogeneity test, one-way analysis of variance was used; for measurement data that do not conform to the normal distribution, non-parametric tests were used; for count data, chi-square test was used; and for pairwise comparison between multiple groups, SNK method was used. The statistical test level P of the whole article was 0.05. |
| Research Results Publication Form | Academic paper |

## I. Research Objective

**Primary Objective**: This study aims to observe and compare the safety, effectiveness, and impact on patient prognosis of the double-cuff bronchial blocker and single-cuff bronchial blocker in thoracoscopic lobectomy, so as to provide a basis for selecting appropriate airway management tools during one-lung ventilation in clinical practice.

## II. Research Background

The purpose of lung isolation technology is to provide surgeons with the largest possible operating space and surgical field of view while avoiding contamination of the surgical side of the lung by tumors, sputum, secretions, etc. in the ipsilateral lung. In recent years, bronchial blockers have become increasingly popular in thoracic surgery due to their advantages such as simple operation and less trauma. However, the single-cuff bronchial blocker has disadvantages such as a high displacement rate and inability to isolate the surgical lobe from the non-surgical lobe on the surgical side. The double-cuff bronchial blocker retains the advantages of the single-cuff bronchial blocker and increases the anchoring point, realizing the isolation of the surgical lobe from the non-surgical lobe on the surgical side. However, its clinical application effect still needs to be verified. Therefore, this study aims to explore the application effects of the two blockers in thoracoscopic lobectomy and provide a reference for selecting an appropriate lung isolation method in clinical practice.

## III. Experimental Basis

**Animal experiments and literature basis in the early stage of the study**: This study refers to the research of Zou Gongsheng et al. "Comparison of Bronchial Blocker and Double-Lumen Bronchial Tube in One-Lung Ventilation", Li Shanshan et al. "Comparison of the Application of Bronchial Blocker and Double-Lumen Bronchial Tube in Esophageal Cancer Radical Resection", and Young Yoo J et al. "Disconnection Technique With a Bronchial Blocker for Improving Lung Deflation: A Comparison With a Double-Lumen Tube and Bronchial Blocker Without Disconnection", and determined the research protocol of this study through pilot experiments.

1. **Basis for subject selection**: Referring to the research protocols of previous studies, the patient inclusion criteria of this study were formulated based on relevant ethical principles.

## IV. Research Contents

1. **Test population**: 80 patients who underwent thoracoscopic lung tumor resection with bronchial blocker technology in Nanpi County People's Hospital were selected.
2. **Sample size calculation**: This study adopts a completely randomized design. According to the results of literature retrieval, the required sample size was estimated by comparing two sample rates (α = 0.05, 1 - β = 0.8), and the sample size of each group was 32. Considering the possible loss to follow-up, operation failure, and withdrawal from the trial, 40 patients were included in each group, and the total sample size was 80.
3. **Specific research contents**:
   3.1 **Basic preparation**
   All patients fasted for 8 hours and abstained from drinking for 2 hours before surgery. The operating room nurse established an upper limb venous access, and the anesthesiologist connected the IntelliVue MP50 monitor to record the pulse, oxygen saturation (SpO2), and electrocardiogram (ECG). Radial artery catheterization was performed under local anesthesia, and the ambulatory blood pressure (ABP) was monitored. Atropine 0.5 mg was intravenously injected 5 minutes before the start of anesthesia.
   3.2 **Anesthesia induction**
   The patient was oxygenated and denitrogenated under the face mask. Sufentanil 0.2 - 0.4 μg/kg, etomidate 0.2 - 0.3 mg/kg, and cisatracurium 0.2 mg/kg were injected intravenously in sequence. After the patient lost consciousness, the face mask was used for assisted ventilation for 3 minutes. In group D, an 8.0# single-lumen endotracheal tube was inserted by an experienced anesthesiologist under direct vision through the mouth, and the cuff was placed 2 cm below the glottis. At the same time, a double-cuff bronchial blocker was inserted and positioned in the appropriate position of the ipsilateral lung under the guidance of a fiberoptic bronchoscope (for right lung surgery, the double cuffs were placed in the right main bronchus and the intermediate bronchus respectively; for left lung surgery, the double cuffs were placed in the left main bronchus and the left lower lobe bronchus respectively). The endotracheal tube and the double-cuff bronchial blocker were fixed. In group S, an 8.0# single-lumen endotracheal tube was inserted by an experienced anesthesiologist under direct vision through the mouth, and the cuff was placed 2 cm below the glottis. Then, a 9Fr single-cuff bronchial blocker was inserted into the ipsilateral main bronchus through the single-lumen tube. After confirming the good position of the blocker with a fiberoptic bronchoscope, it was fixed. The ventilator was used to control breathing, and the airway peak pressure (Ppeak) and end-tidal carbon dioxide partial pressure (PetCO2) were monitored.
   3.3 **Anesthesia maintenance**
   The anesthesia of all patients during the operation was maintained by a combined intravenous-inhalation anesthesia of remifentanil and sevoflurane. Remifentanil (0.05 - 0.5 μg/kg/min) was continuously pumped by a micro-infusion pump, and sevoflurane (1 - 3%) was inhaled at the same time to maintain the bispectral index (BIS) between 40 and 60. Cisatracurium 0.05 mg/kg was additionally injected every half an hour. During two-lung ventilation, the respiratory parameters were set as follows: tidal volume (Vt) 8 - 10 ml/kg, respiratory rate (f) 12 breaths/min, inspiratory-expiratory ratio (I:E) 1:2. The fresh gas flow rate was 2 L/min, and the inspired oxygen concentration (FiO2) was 100%. The end-tidal carbon dioxide partial pressure (PetCO2) was maintained between 35 and 45 mmHg. During one-lung ventilation, the respiratory parameters were set as follows: tidal volume 6 - 8 ml/kg, respiratory rate 15 breaths/min, inspiratory-expiratory ratio 1:2, PEEP 5 cmH2O. The fresh gas flow rate was 2 L/min, and the inspired oxygen concentration (FiO2) was 80%. The end-tidal carbon dioxide partial pressure (PetCO2) was maintained between 35 and 45 mmHg.
   3.4 **Experimental grouping and treatment**
   A doctor not involved in this experiment used a computer program to randomly divide 80 patients into 2 groups.
   Group D (double-cuff bronchial blocker group): In group D, an 8.0# single-lumen endotracheal tube was inserted by an experienced anesthesiologist under direct vision through the mouth, and the cuff was placed 2 cm below the glottis. At the same time, a double-cuff bronchial blocker was inserted and positioned in the appropriate position of the ipsilateral lung under the guidance of a fiberoptic bronchoscope (for right lung surgery, the double cuffs were placed in the right main bronchus and the intermediate bronchus respectively; for left lung surgery, the double cuffs were placed in the left main bronchus and the left lower lobe bronchus respectively). The endotracheal tube and the double-cuff bronchial blocker were fixed.
   Group S (single-cuff bronchial blocker group): In group S, an 8.0# single-lumen endotracheal tube was inserted by an experienced anesthesiologist under direct vision through the mouth, and the cuff was placed 2 cm below the glottis. Then, a 9Fr single-cuff bronchial blocker was inserted into the ipsilateral main bronchus through the single-lumen tube. After confirming the good position of the blocker with a fiberoptic bronchoscope, it was fixed.
4. **Measured and monitored indicators**
   4.1 Record the general information of the patients, including gender, age, height, and weight.
   4.2 Continuously monitor ABP, SpO2, and PetCO2 during the operation.
   4.3 Record the insertion time of the blocker, lung collapse grade, incidence of hypoxemia, type and duration of surgery, one-lung ventilation time, surgeon satisfaction, number of displacements of the bronchial blocker, contamination rate of the non-surgical lobe of the ipsilateral lung under the fiberoptic bronchoscope, tracheal wall injury under the fiberoptic bronchoscope, and incidence of adverse cardiovascular events.
   4.4 Record SpO2 and end-tidal carbon dioxide partial pressure (PetCO2) at the following time points; partial pressure of carbon dioxide (PaCO2), partial pressure of oxygen (PaO2), pH, and lactate (Lac) in arterial blood gas.
   T0: Before surgery for all patients;
   T1: 30 minutes after one-lung ventilation;
   T2: 60 minutes after one-lung ventilation;
   4.5 One day after surgery, record the occurrence of postoperative pulmonary complications such as pneumonia and atelectasis according to the chest X-ray.
   4.6 Calculate the hospital stay and hospitalization costs of the patients.
   4.7 The main research indicator is: the number of displacements of the bronchial blocker.
   The secondary research indicators are: insertion time of the blocker, lung collapse grade, incidence of hypoxemia, surgeon satisfaction, tracheal wall injury under the fiberoptic bronchoscope, contamination rate of the non-surgical lobe of the ipsilateral lung under the fiberoptic bronchoscope, incidence of adverse cardiovascular events, blood gas analysis indicators at different time points, and occurrence of postoperative pulmonary complications such as pneumonia and atelectasis one day after surgery.

## V. Research Methods

1. **Inclusion criteria (diagnostic criteria, inclusion criteria, exclusion criteria)**
   **Inclusion Criteria**
   (1) 80 patients who were scheduled to undergo thoracoscopic lobectomy in our hospital from September to December 2024 were selected;
   (2) American Society of Anesthesiologists (ASA) physical status classification was grade I - II;
   (3) Age 18 - 65 years old, body mass index (BMI) 18.5 - 25 kg/m²;
   (4) No obvious abnormality in lung function, forced expiratory volume in one second/forced vital capacity (FEV1/FVC%) > 70%, no restrictive or obstructive ventilatory dysfunction;
   (5) No obvious abnormality in cardiac function, no cardiovascular disease, ejection fraction (EF) > 50%;
   (6) No anemia or other hematological diseases before surgery and no history of radiotherapy or chemotherapy;
   (7) Agree to participate in this study and sign the informed consent form.
   **Exclusion Criteria**
   (1) The patient or their family members refuse to participate in the trial;
   (2) Moderate or severe abnormality in cardiopulmonary function;
   (3) History of bronchial asthma and airway hyperresponsiveness;
   (4) Patients with pulmonary infection, bronchopleural fistula, emphysema, or bullae;
   (5) The length of the right main bronchus is less than 0.5 cm;
   (6) Patients with contraindications for using the blocker, such as tumors in the airway.
2. **Subject grouping**: A doctor not involved in this experiment used a computer program to randomly divide 80 patients into 2 groups. Group D (double-cuff bronchial blocker group): After anesthesia induction and intubation, an 8.0# single-lumen endotracheal tube was inserted by an experienced anesthesiologist under direct vision through the mouth, and the cuff was placed 2 cm below the glottis. At the same time, a double-cuff bronchial blocker was inserted and positioned in the appropriate position of the ipsilateral lung under the guidance of a fiberoptic bronchoscope (for right lung surgery, the double cuffs were placed in the right main bronchus and the intermediate bronchus respectively; for left lung surgery, the double cuffs were placed in the left main bronchus and the left lower lobe bronchus respectively). The endotracheal tube and the double-cuff bronchial blocker were fixed. Group S (single-cuff bronchial blocker group): After anesthesia induction and intubation, an 8.0# single-lumen endotracheal tube was inserted by an experienced anesthesiologist under direct vision through the mouth, and the cuff was placed 2 cm below the glottis. Then, a 9Fr single-cuff bronchial blocker was inserted into the ipsilateral main bronchus through the single-lumen tube. After confirming the good position of the blocker with a fiberoptic bronchoscope, it was fixed.
3. **Subjects' early withdrawal/termination of the trial criteria**: All the required experimental design data have been collected; intraoperative hypoxemia (SPO2 ≤ 92%) occurs in the patient; intraoperative massive hemorrhage, allergic reaction, arrhythmia and other unexpected situations occur.

### VI. Subject Management

1. Recruitment method of subjects: Select patients who will undergo thoracoscopic lung tumor resection with bronchial blocker technology in Nanpi County People's Hospital.
2. Informed consent process: During the preoperative anesthesia visit, explain the content of the informed consent form in detail to the patient and their legal representatives, and obtain their consent and signatures.

### VII. Start and End of the Trial: September 1, 2024 - December 31, 2024

### VIII. Data Security and Monitoring Plan

1. Overview of data management methods:
   Data management: Enter the data of various indicators collected in the experiment, using the double-entry method.
   Data quality control: Data supervisors do not participate in the implementation of the experiment and are independent of the sponsor without any conflict of interest. Data supervisors are responsible for verifying the entered data and auditing random labels, groupings, follow-up records, and data collection.
2. Reporting and collection of adverse events and serious adverse events
   The drugs, consumables, and equipment involved in this study are all required for thoracoscopic lung surgery. Therefore, the risks involved are the same as those of conventional thoracic general anesthesia, and the main risks are hypoxemia, circulatory function suppression, etc. If hypoxemia occurs, that is, the blood oxygen saturation is lower than 90%, the anesthesiologist can take corresponding measures according to the patient's and surgical conditions, including: applying PEEP to the healthy lung, increasing the CPAP pressure on the surgical side, stopping one-lung ventilation and changing to two-lung ventilation, etc. Generally, hypoxemia will be relieved within 1 - 2 minutes after taking corresponding measures. If circulatory function suppression occurs, vasopressors, atropine, etc. will be used as appropriate.
3. Medical safety measures: The anesthesia process of all patients is monitored by two anesthesiologists throughout the process, including at least one attending physician or above, to ensure patient safety.
4. Internal analysis plan of data: After the end of the experiment, the data will be handed over to a statistician for statistics.
5. Frequency of submitting data security and monitoring reports to the ethics committee: After the end of the experiment, the data will be handed over to the ethics committee.

### IX. Compliance with Ethical Principles and Relevant Regulations: This study complies with ethical principles and relevant regulations.

### X. Statistical Analysis Plan: Use statistical software SPSS21.0 version for analysis. For measurement data that meet the comparison of means of single-factor multiple samples and pass the normality test and variance homogeneity test, one-way analysis of variance is used; for measurement data that do not conform to the normal distribution, non-parametric tests are used; for count data, chi-square test is used; and for pairwise comparison between multiple groups, SNK method is used. The statistical test level P of the whole article is 0.05.

### XI. Publication Form of Research Results: Report the research results through publishing articles.
