# Supplementary material for: Double-cuff versus single-cuff bronchial blockers for video-assisted thoracoscopic lobectomy: A randomized controlled trial
Source: PLoS One. 2026 May 21;21(5):e0350007. doi: 10.1371/journal.pone.0350007 (PMC13193552; doi:10.1371/journal.pone.0350007)
Supplement: Attachment — Submitted filename: Response to Reviewers.docx [file pone.0350007.s004.docx]

List of Responses

Responds to Journal Requirements:

*Q1:* *Please ensure that your manuscript meets PLOS ONE's style requirements, including those for file naming. The PLOS ONE style templates can be found at https://journals.plos.org/plosone/s/file?id=wjVg/PLOSOne_formatting_sample_main_body.pdf and* [*https://journals.plos.org/plosone/s/file?id=ba62/PLOSOne_formatting_sample_title_authors_affiliations.pdf*](https://journals.plos.org/plosone/s/file?id=ba62/PLOSOne_formatting_sample_title_authors_affiliations.pdf)*.*

Response: We have checked the manuscripts and file naming to ensure compliance with PLOS ONE's style requirements. If there are still errors, please let us know.

*Q2: We note that the grant information you provided in the ‘Funding Information’ and ‘Financial Disclosure’ sections do not match. When you resubmit, please ensure that you provide the correct grant numbers for the awards you received for your study in the ‘Funding Information’ section.*

Response: We have carefully checked and revised the Funding Information section, and we confirm that the information filled in this section is accurate and complete. The correct grant number 20240190 (Health Commission of Hebei Province) has been accurately entered in the Funding Information section for this study.

*Q3:* *Please note that funding information should not appear in any section or other areas of your manuscript. We will only publish funding information present in the Funding Statement section of the online submission form. Please remove any funding-related text from the manuscript.*

Response: We have removed all funding-related text from the main text and other sections of the manuscript. All funding information is now only presented in the Funding Statement section of the online submission form, in full compliance with your requirement.

*Q4:* *We note that you have a patent relating to material pertinent to this article. Please provide an amended statement of Competing Interests to declare this patent (with details including name and number), along with any other relevant declarations relating to employment, consultancy, patents, products in development or modified products etc. Please confirm that this does not alter your adherence to all PLOS ONE policies on sharing data and materials, as detailed online in our guide for authors http://journals.plos.org/plosone/s/competing-interests by including the following statement: "This does not alter our adherence to PLOS ONE policies on sharing data and materials.” If there are restrictions on sharing of data and/or materials, please state these. Please note that we cannot proceed with consideration of your article until this information has been declared. This information should be included in your cover letter; we will change the online submission form on your behalf.*

Response: We have submitted the amended Competing Interests statement in our cover letter, which declares the relevant patent: Patent name: A Double-cuff bronchial blocker; Patent number: ZL202121177425.1. We also declare no other relevant conflicts relating to employment, consultancy, products in development or modified products. The statement includes the following sentence: “This does not alter our adherence to PLOS ONE policies on sharing data and materials.” No restrictions apply to the sharing of data and materials in this study. We appreciate your assistance in updating the online submission form with this information.

*Q5:* *Please include captions for your Supporting Information files at the end of your manuscript, and update any in-text citations to match accordingly. Please see our Supporting Information guidelines for more information:* [*http://journals.plos.org/plosone/s/supporting-information*](http://journals.plos.org/plosone/s/supporting-information)*.*

Response: We confirm that no Supporting Information files are included or cited in this manuscript. Therefore, there are no Supporting Information captions to be added at the end of the manuscript, and no in-text citations to Supporting Information require updating.

*Q6: Thank you for providing your underlying data as Supporting Information. We note that the data set contains text or data that is not in English. Please note that PLOS is an English-language publisher, so we require data sets to be provided in English as well. Please upload an English-language version of your data set. This will also allow us to determine if your data follows PLOS standards per our Data Availability policy here:* [*https://journals.plos.org/plosone/s/data-availability*](https://journals.plos.org/plosone/s/data-availability)*.*

Response: We have translated all non-English text and data in the original dataset into English and uploaded the revised English-language version of the data set as Supporting Information. The data now fully complies with PLOS ONE’s language and Data Availability standards.

*Q7:* *If the reviewer comments include a recommendation to cite specific previously published works, please review and evaluate these publications to determine whether they are relevant and should be cited. There is no requirement to cite these works unless the editor has indicated otherwise.*

Response: No citation request was made by the reviewer.

Responds to the reviewer's comments:

Reviewer ＃1:

*Q1:* *Randomization and Allocation Concealment: The use of a computer-generated random number table to randomly assign patients is documented. However, there is no description of how the randomization sequence was concealed until the time of assignment (e.g., whether opaque, sealed envelopes with sequential numbers were used, or whether a central registration system was employed). This is an important item in the CONSORT* *statement. Please specify.*

Response: We appreciate your constructive suggestion. We have supplemented the manuscript with a detailed description of the random sequence generation, allocation concealment method, and the timing at which anesthesiologists and surgeons were informed of group assignment, in full accordance with the CONSORT statement requirements.

Changes (page 6, line 43-54): “After enrollment into the trial, all patients were randomly divided into two groups…”

*Q2: Statistical Analysis:2-1. Lines 114-117:* *Because the number of patients who experienced these events was low, Fisher's exact test should be used instead of the chi-square test.*

Response: We appreciate your constructive suggestion. The chi‑square test was retained for grades of lung collapse and surgeon satisfaction, while Fisher’s exact test was used for all other relevant categorical indicators in the revised manuscript.

Changes (page 9, line 127-130): “Malposition rates (%) were compared using Fisher exact tests…”

*Q3: 2-2. Table 2: In the "Difference" column, please specify the point estimate in addition to the 95% CI. Furthermore, in the "Risk ratio" column, the calculated values seem to represent the Odds Ratio rather than the Risk Ratio. Please clarify and correct this.*

Response: We appreciate your constructive suggestion. We have supplemented the **point estimate** in the “Difference” column of Table 2, revised the “Risk ratio” column to **Odds Ratio (OR)** uniformly, and recalculated the OR values and corresponding 95% confidence intervals (CI). The definitions of these indicators have been clearly stated in the table footnote, and all relevant content has been updated accordingly.

*Q4: Table 3: In the "Risk ratio" column, the calculation seems to be based on the number of patients who did NOT experience the events. Please correct this to reflect the occurrence of the events.*

Response: We appreciate your constructive suggestion. We have recalculated the relevant OR values in Table 3 based on the number of events to replace the original results. Meanwhile, we have added the Fisher’s exact test results and 95% CI for indicators with low event counts. All tables have been updated accordingly.

*Q5: Language and Formatting Issues*

*The manuscript requires thorough language editing. Here are specific examples of errors that need to be corrected:*

*5-1. Abstract (Methods): The sentence "we enrolled and randomized 80 patients..." begins with a lowercase "w".*

*5-2 Abstract (Methods): There is a missing period before "The primary outcomes": "...(ScBB used during one-lung ventilation) The primary outcomes..."*

*5-3* *Abstract (Results): There is an unnecessary comma in: "...significantly lower in the DcBB, than the ScBB group..."*

*5-4 Abstract (Results): There is a missing period before "However": "...in the DcBB cohort (P=0.002) However, positioning the DcBB...".*

*5-5 Line 131: There is a missing period between sentences: "Between-group differences were not significant The duration of BB placement...".*

*5-6* *Lines 139-150: The text contains a major structural issue that leaves sentences disjointed and garbled. For example, a sentence cuts off with "The malposition", continues awkwardly on the next line, and later abruptly inserts fragments like "rate of the DLT is lower than that of BBs¹s and a meta-analysis also found a rate of 17.0%,14 which was" in the middle of a different thought. This entire paragraph needs to be rewritten for coherence.*

*5-7 Line 154: There is a typo in the word "skills": "...in terms of bronchoscopic skilss...".*

*5-8* *Table 3: There is a typo in the variable name: "Contamination rate of non-surgica" instead of "non-surgical".*

Response: We appreciate your constructive suggestion. We have carefully revised all grammatical and formatting errors throughout the manuscript, including correcting capitalization, adding missing periods, deleting redundant commas, and fixing spelling mistakes (e.g., “skilss” corrected to “skills”). In addition, we have completely rewritten the disorganized paragraphs on lines 139–150 to ensure logical coherence. All linguistic revisions have been updated in the revised manuscript.

Reviewer #2:

*Q1:* *Was an intent to treat approach used?*

Response: We appreciate your constructive comment. This study was analyzed using the intention-to-treat principle. All 80 patients who were enrolled and randomized were included in the final statistical analysis. No patient was lost to follow-up, withdrew, or had protocol deviation. All statistical analyses (including primary and secondary endpoints) were based on the full dataset of these 80 patients without any exclusion, which preserved the balance of randomization. We have supplemented this description in the statistical analysis section of the revised manuscript.

Changes (page 6, line 43-54 ): “ After enrollment into the trial, all patients were randomly divided into two groups…”

*Q2: Please provide details of randomization. When were the anesthesiologist and the surgeon informed about the allocation? How was allocation concealed until then?*

Response: Thank you for your suggestion. We have supplemented the manuscript with a detailed description of the random sequence generation, allocation concealment method, and the timing at which anesthesiologists and surgeons were informed of group assignment, in full accordance with the CONSORT statement requirements.

Changes (page 6, line 43-54 ): “ After enrollment into the trial, all patients were randomly divided into two groups…”

*Q3: Please specify how the groups were so equally balanced? Was blocking used? If not, please give more details about the allocation.*

Response: We appreciate your constructive comment. The high degree of balance in baseline characteristics between the two groups in this study can be attributed to the strict randomization scheme and restrictive inclusion criteria. First, a 1:1 simple randomization was used to ensure balanced allocation of sample size. Second, strict restrictions were applied to age, BMI, ASA classification, pulmonary function, and other indicators in the inclusion criteria, which reduced potential baseline confounding factors. Stratified block randomization was not adopted in this study because the baseline characteristics of the enrolled population were relatively homogeneous, and no additional stratification was necessary. We have supplemented the above explanation in the revised manuscript.

*Q4:* *Details of randomization deserves its own section.*

Response: We appreciate your constructive suggestion. We have described the protocol for random sequence generation in detail and presented it clearly in the Participants subsection under the Methods section in the revised manuscript.

*Q5:* *Please remove p values from Table 1.*

Response: We appreciate your constructive suggestion. We have removed the P-values for all variables in Table 1, retaining only the group labels and the numerical values for demographic and baseline clinical characteristics.

*Q6: Please standardize number of decimal places in tables.*

Response: We appreciate your constructive suggestion. The number of decimal places for all tables has been standardized to two decimal places throughout the revised manuscript.

*Q7:* *Please present p values or CIs in Table 4.*

Response: We appreciate your constructive suggestion. We have added two additional columns in Table 4 (blood gas analysis) for P-values (between-group comparison) and 95% CIs (mean difference) at the end of the table.

*Q8:* *Please describe the flow diagram in the results section.*

Response: We appreciate your constructive suggestion. We have described the CONSORT flow of the present study in detail in the Results section (82 patients screened, 2 excluded, 80 enrolled, and no loss to follow-up).

Changes (page 11, line 151-156): “A total of 82 patients with lung cancer scheduled…”

*Q9: Would a GEE that took into account the multiple measures for arterial partial pressure of oxygen and CO2 be useful?*

Response: We appreciate your constructive suggestion. For repeated blood gas parameters including PaO2 and PaCO2, statistical analysis was performed using generalized estimating equations (GEE), incorporating the interaction effect between time and group. This method is more appropriate for between-group comparisons of repeated measurement data. The relevant statistical methodology has been supplemented in the Analysis subsection, and the statistical results have been updated in Table 4.

Changes (page 10, line 134-136 ): “Continuous variables with repeated measurements…”

*Q10:* *A careful reading for grammar, especially verb tense, is necessary.*

Response: We appreciate your constructive suggestion. The entire manuscript has been carefully checked and revised for grammatical issues, with particular attention paid to verb tense consistency. All relevant linguistic corrections have been implemented in the revised manuscript. The manuscript has undergone professional language editing by a certified editing service, ensuring that all grammatical, tense, spelling, and formatting issues noted are fully addressed.

Reviewer #3:

*Q1:* *A critical limitation of this study is that both anesthesiologists and surgeons were aware of the allocated intervention during the procedure. The manuscript explicitly states that the operators could easily determine which bronchial blocker was used and therefore were aware of group allocation. Because the primary endpoint (malposition) is largely dependent on intraoperative clinical judgment and bronchoscopy assessment by the treating anesthesiologist, the absence of blinding introduces a substantial risk of performance and detection bias. In device trials where operator technique strongly influences outcomes, lack of blinding severely limits the reliability of comparative conclusions. Consequently, the study design does not sufficiently support the causal inference that the double-cuff device reduces malposition.*

Response: We greatly appreciate your rigorous and valuable comment on the potential bias related to outcome assessment and blinding. We have carefully addressed your concern with comprehensive revisions and additional explanations as follows: First, the diagnostic criteria for bronchial blocker malposition were strictly established according to previous well-recognized and widely cited literature (Reference 9 in the manuscript). The definitions and assessment criteria used in our study are consistent with those in mainstream clinical studies of bronchial blockers, ensuring the objectivity and reproducibility of the primary endpoint. Second, all malposition events were objectively confirmed by fiberoptic bronchoscopy. Whenever malposition was suspected intraoperatively, we immediately performed bronchoscopy to record the exact position and stored bronchoscopic images or videos as reliable objective evidence. All outcomes were determined based on these visual records rather than on subjective judgment, which greatly reduced detection bias. We have also supplemented these detailed procedures in the revised manuscript. Third, due to the obvious structural differences between double-cuff and single-cuff devices, blinding of anesthesiologists and surgeons was not feasible in this study. We have explicitly stated this limitation in the revised Discussion section. We hope these revisions and clarifications adequately address your concerns.

Changes (page 8, line 94-100 ): “Intraoperatively, a malpositioned BB was considered…”

*Q2: The primary endpoint—bronchial blocker malposition—is not rigorously defined. In this study, malposition was considered when airway pressure increased, ventilation could not be maintained, or the surgical lung suddenly inflated, followed by confirmation using bronchoscopy. These criteria are nonspecific and may occur for multiple intraoperative reasons unrelated to blocker displacement. Moreover, the decision to perform bronchoscopy and confirm malposition appears to rely on the judgment of the anesthesiologist who was aware of the device type. Without predefined objective bronchoscopic criteria or independent adjudication, the validity and reproducibility of the primary endpoint remain questionable.*

Response: Thank you for your additional comment regarding potential performance and detection bias in this study. We fully agree with your professional comment and have taken rigorous measures to address this issue, consistent with our response to your first comment. First, the diagnostic criteria for bronchial blocker malposition were strictly established based on previously published and validated literature, ensuring the objectivity and consistency of outcome assessment. Second, all malposition events were intraoperatively verified by fiberoptic bronchoscopy and documented with electronic images. Postoperatively, all images were independently reviewed and confirmed by a second anesthesiologist who was not involved in the anesthesia procedure and was blinded to group allocation, which effectively minimized subjective judgment and detection bias. Third, due to the substantial structural differences between double‑cuff and single‑cuff bronchial blockers, blinding of anesthesiologists and surgeons was not feasible in this study. We have clearly stated this limitation in the Discussion section and proposed that future multi‑center trials using visually identical devices could be designed to enable double‑blind protocols, which would further reduce potential bias. We believe these measures and revisions have adequately addressed the bias-related concerns and enhanced the methodological rigor of this study.

*Q3:* *Several authors are listed as inventors of the investigated double-cuff bronchial blocker and hold a related patent. While such involvement does not necessarily invalidate the research, it increases the importance of strict methodological safeguards such as independent outcome adjudication or blinded assessment. These safeguards are not present in this study, which further raises concerns about bias.*

Response: Thank you for your insightful comment regarding potential investigator bias related to the inventor of the double‑cuff bronchial blocker. We fully appreciate this crucial point and have implemented strict measures to avoid any relevant bias as follows: First, the entire clinical implementation, intraoperative management, data collection, and original recordation of this study were independently performed by Dr. Guoliang Tang and Dr. Xiaobin Li, who had no involvement in the invention or design of the double‑cuff bronchial blocker. Second, the secondary verification of bronchial blocker malposition was independently conducted by another senior anesthesiologist who also did not participate in the device invention and was blinded to group allocation, ensuring objective outcome assessment. Third, the inventor of the double‑cuff bronchial blocker, as one of the authors, only contributed to the initial study design and the academic writing of this manuscript. The inventor did not participate in patient recruitment, intraoperative management, data collection, outcome assessment, statistical analysis, or result interpretation—all of which might introduce potential bias. All data processing and statistical analyses were conducted independently by investigators with no financial or institutional interest in the device. We have also added a supplementary explanation in the revised manuscript.

Changes (page 10, line 143-147 ): “To minimize investigator bias related to the inventor…”

*Q4:* *Although a statistical difference in malposition incidence was reported, the study failed to demonstrate meaningful differences in clinically relevant secondary outcomes such as hypoxemia, surgical duration, lung collapse quality, or postoperative complications. Therefore, even if the reported reduction in malposition were valid, the clinical benefit of the device remains unclear.*

Response: We appreciate your insightful comment regarding the clinical significance of the primary endpoint and the non-significant results of secondary endpoints. We have carefully elaborated the relevant rationales as follows: The primary endpoint of this study was the incidence of bronchial blocker malposition. In clinical practice, any malposition of the blocker requires immediate endoscopic adjustment by the anesthesiologist to restore effective one-lung ventilation. A lower malposition rate in the DcBB group directly reduces the frequency of intraoperative endoscopic manipulations, which not only decreases the anesthesiologist’s intraoperative workload but also minimizes mechanical irritation and potential trauma to the patient’s tracheobronchial mucosa. This reduction in procedural interventions is clinically meaningful for enhancing the stability of intraoperative ventilation and lowering the risk of airway-related complications, which is the core clinical value of this study. The non-significant between-group differences in secondary outcomes (e.g., hypoxemia, adverse cardiovascular events, pulmonary infiltration on POD 1) can be explained by the following aspects:

1. The anesthesiologists maintained real-time monitoring of the blocker position and ventilation status throughout the operation. Any occurrence of malposition was identified immediately and corrected promptly via fiberoptic bronchoscopy. This timely intervention prevented the deterioration of ventilation efficiency, thus avoiding the occurrence of hypoxemia and subsequent adverse cardiovascular events and eliminating the impact of transient malposition on postoperative pulmonary outcomes.

2. The sample size of this study was rigorously calculated according to the incidence of bronchial blocker malposition (the primary endpoint), aiming to ensure adequate statistical power for the primary comparison. Secondary endpoints were designed as exploratory indicators; however, the current sample size may be insufficient to detect potential between-group differences for these indicators. Larger-sample, multi-center studies are warranted to verify the clinical benefits of DcBB on these secondary outcomes.

3. All patients received uniform intraoperative ventilation strategies, anesthetic protocols, and postoperative care. This strict, standardized management further reduced the variability of secondary outcomes, contributing to the non-significant between-group differences.

Changes (page 12, line 180-193 ): “ In our study, the lower malposition rate with the…”

*Q5:* *The authors conclude that the double-cuff bronchial blocker may be a superior airway management tool. However, the data do not convincingly demonstrate superiority. In fact, device positioning required significantly longer time in the DcBB group. Furthermore, the mechanistic rationale for why an additional cuff should substantially reduce malposition is not convincingly supported by either experimental data or prior literature.*

Response: We sincerely appreciate your insightful and rigorous comments. We have carefully revised the manuscript and addressed your concerns as follows: We have removed all statements implying that the DcBB is “superior” and have revised both the abstract and conclusion to be fully objective and evidence-based. The conclusion now states: “In patients undergoing thoracoscopic lobectomy, the DcBB was associated with a lower incidence of malposition than the ScBB. The DcBB did not increase tracheal wall injury or cause significant adverse hemodynamic effects, thus providing a new alternative for airway management during thoracoscopic lobectomy.” This revision avoids overstatement and simply presents the findings as a new option for clinical practice.

We agree that the precise mechanism by which an additional cuff reduces malposition remains incompletely understood. As you noted, current literature does not provide a definitive mechanistic explanation for the reduced malposition of either double-lumen tubes or double-cuff blockers. Most interpretations are based on clinical observations and reasonable inference rather than conclusive evidence. We have moderated the relevant description in the Discussion section to reflect this uncertainty and maintain scientific caution.

To further verify the mechanical stability of the double-cuff design, we have performed an ex vivo experimental study comparing resistance to displacement between DcBB and ScBB. Preliminary results indicate that the force required to dislocate the DcBB is more than 1.2 times greater than that for the ScBB, supporting improved stability of the double-cuff structure. This ex vivo study is currently being prepared for submission as a separate manuscript. Should the reviewer or editor be interested in these supplementary data, we are willing to provide detailed information via email.

Changes (page 14, line 210-213 ): “ In patients undergoing thoracoscopic lobectomy…”

*Q6: The sample size calculation is also questionable. The authors assumed a malposition rate of 40% in the single-cuff BB group, even though the single-cuff blocker represents a standard and widely used device in current practice. This assumption appears inflated and insufficiently justified. An overestimated control event rate can artificially reduce the required sample size and make the investigational device appear more favorable. Therefore, the statistical planning itself raises concern regarding the internal validity of the trial.*

Response: We sincerely appreciate your careful and rigorous comment on the assumed malposition rate in the single‑cuff bronchial blocker (ScBB) group for our sample size calculation. We have verified this assumption using high‑quality published evidence and clarify as follows:

A large meta‑analysis published in 2023 reported a pooled malposition rate of 31.9% for conventional bronchial blockers during thoracic surgery [1].

A recent clinical study demonstrated that the dislocation rate of double‑lumen tubes (DLTs) reached 44.6% after patient positioning from the supine to the lateral decubitus position [2]. It is well established that the malposition rate of bronchial blockers is higher than that of DLTs in clinical practice.

A recently registered randomized controlled trial protocol investigating bronchial blockers also adopted a malposition rate of 40% as the baseline for sample size calculation, which is fully consistent with our assumption [3].

Therefore, our assumption of a 40% malposition rate in the ScBB group is consistent with real‑world clinical data and recent published evidence, rather than an inflated or unjustified estimation. This assumption is clinically reasonable and supports the internal validity of our sample size calculation. We have added these relevant literature citations in the revised manuscript to further justify the sample size estimation.

[1] Palaczynski P, Misiolek H, Szarpak L, Smereka J, Pruc M, Rydel M, et al. Systematic review and meta-analysis of efficiency and safety of double-lumen tube and bronchial blocker for one-lung ventilation. J Clin Med. 2023;12: 1877. doi: 10.3390/jcm12051877.

[2] Chen ZY, Lin YM, Wu JH, Fu YY, Xu XT, Li Y, et al. Does the periportal end of a double-lumen endobronchial tube need to be fixed to prevent dislocation of the cuffed end caused by a change in position? A randomized controlled trial. Ann Med. 2023;55(2): 2247422. doi: 10.1080/07853890.2023.2247422.

[3] Zhang Z, Liu X, Zhang X, Zhou B, Tang Y, Tong F, et al. Study protocol for a randomized controlled trial assessing the effect of lateral position intubation on bronchial blocker placement during unilateral video-assisted thoracic surgery. Trials. 2025;26(1): 553. doi: 10.1186/s13063-025-09287-7.
