# Supplementary material for: Double-cuff versus single-cuff bronchial blockers for video-assisted thoracoscopic lobectomy: A randomized controlled trial
Source: PLoS One. 2026 May 21;21(5):e0350007. doi: 10.1371/journal.pone.0350007 (PMC13193552; doi:10.1371/journal.pone.0350007)
Supplement: Attachment — Submitted filename: Response_to_Reviewers_auresp_2.docx [file pone.0350007.s005.docx]

List of Responses

Response to Reviewers' Comments:

Reviewer ＃1:

*Q1: The statement “P > 0.05” in the Results section of the main text does not add any meaningful information and should be removed.*

Response: We fully agree and have **deleted all redundant “P > 0.05” statements** in the Results section. Only clinically meaningful significance descriptions are retained.

Changes (page 12, line 234-235): “did not significantly differ between the groups (Table 3)…”

*Q2: The estimated odds ratio for the “Contamination rate of non-surgical” in Table 3 appears not to have converged. It would be more appropriate to report this as “Not estimated.”*

Response: We have corrected Table 3: the odds ratio for contamination rate is now reported as “**Not estimated**” as suggested.

Changes (Table 3): 0.00 (0.00, ∞) →**Not estimated.**

*Q3: The P-value for the between-group comparison at T0 in Table 4 should be removed*.

Response: We have **removed the P-value for T0** in Table 4 for both PaO_2_ and PaCO_2_ as recommended.

Reviewer #3:

*Q1:* *You appropriately acknowledge that blinding of operators was not feasible due to the nature of the devices. However, this limitation is particularly important in the context of this study, as both the intervention and outcome assessment are operator-dependent. The lack of blinding, combined with the subjective components of outcome detection, continues to pose a significant threat to internal validity.*

Response: Thank you for this important and insightful comment. We fully agree that blinding of the anesthesiologists performing device placement and the surgeons conducting the operation was not feasible due to the distinct structural differences between the double‑cuff and single‑cuff bronchial blockers.

However, we would like to emphasize that the primary outcome (malposition incidence) was strictly objective and assessed under blinded conditions. All suspected malposition events were confirmed by bronchoscopic imaging, and all images were independently reviewed and verified by a senior anesthesiologist who was completely blinded to group assignment. Bronchoscopy is a well‑recognized, objective gold standard for diagnosing malposition of airway devices and has been widely adopted in previous relevant studies [1,2].

Furthermore, the diagnostic criteria for malposition (sudden elevation of airway pressure, inability to maintain effective ventilation, or sudden re‑inflation of the surgical lung) are well‑established, objective, and widely accepted in the literature, leaving little room for subjective judgment.

Although some secondary outcomes include subjective components (e.g., lung collapse grading, surgeon satisfaction), our primary conclusion is fully based on the objectively assessed, blinded‑verified primary outcome, which minimizes bias and ensures the internal validity of the core finding.

References:

[1] Patel, M, Wilson, A, Ong, C. Double-lumen tubes and bronchial blockers. BJA EDUC. 2023; 23 (11): 416-424. doi: 10.1016/j.bjae.2023.07.001.

[2] Li, J, Liu, W, Liang, X, et al. Comparing the lung isolation efficacy of bronchial blocker positioning via electromagnetic navigation bronchoscopy versus fiberoptic bronchoscopy: a randomized study. J THORAC DIS. 2024; 16 J THORAC DIS. doi: 10.21037/jtd-24-1516.

[3] Campos J, Kernstine K. A comparison of a left-sided Broncho-Cath with the torque control blocker univent and the wire-guided blocker. Anesth Analg. 2003;96(1): 283-289. doi: 10.1213/00000539-200301000-00056.

*Q2:* *Most importantaly, although additional references have been provided to support the assumed malposition rate in the control group, the chosen assumptions still appear optimistic and may have influenced the estimated effect size and required sample size. This raises concerns regarding the robustness of the statistical planning.*

Response: Thank you for your critical and valuable comment regarding the rationality of our sample size assumptions and the robustness of the statistical planning. We fully appreciate your concern and address the details of our sample size estimation point by point as follows:

First, for the single-cuff bronchial blocker (ScBB) group, we assumed a 40% malposition rate based on solid published clinical evidence. A recent meta-analysis by Palaczynski et al. (2023) reported a pooled malposition rate of 31.9% for bronchial blockers in thoracic surgery [1]. A randomized controlled trial protocol by Zhang et al. (2025) also adopted a 40% malposition rate for the control group during sample size calculation [2]. Notably, a study by Chen et al. (2023) demonstrated that the dislocation rate of double-lumen tubes could reach 44.6% after lateral positioning [3]. It is well recognized that the malposition rate of bronchial blockers is **higher** than that of double-lumen tubes. Therefore, our assumption of 40% for the ScBB group is **not optimistic at all, but rather clinically reasonable.**

For the double-cuff bronchial blocker (DcBB) group, the assumed 12% malposition rate was derived from our preliminary prospective pretest conducted in patients undergoing thoracoscopic lobectomy. This pretest was performed under the same clinical conditions and by the same anesthesiology team as the formal study, guaranteeing its clinical relevance and reliability.

Furthermore, our sample size calculation strictly followed the standard formula for comparing two independent proportions, with a predefined power of 0.8 and a type I error rate of 0.05. We initially calculated that 36 patients per group would be sufficient; to account for potential exclusions or losses to follow-up, we enlarged the sample to 40 patients per group, further improving the robustness of our statistical analysis.

Although the sample size is moderate, our study successfully detected a statistically significant difference in the primary outcome (7.5% vs. 30%, P = 0.02), confirming that the sample size was adequate to identify the expected effect size.

Collectively, these points demonstrate that our sample size assumptions were clinically reasonable, evidence-based, and the statistical design was rigorous and reliable, with a low risk of biased effect size estimation.

References:

[1] Palaczynski P, Misiolek H, Szarpak L, Smereka J, Pruc M, Rydel M, et al. Systematic review and meta-analysis of efficiency and safety of double-lumen tube and bronchial blocker for one-lung ventilation. J Clin Med. 2023;12: 1877. doi: 10.3390/jcm12051877.

[2] Zhang Z, Liu X, Zhang X, Zhou B, Tang Y, Tong F, et al. Study protocol for a randomized controlled trial assessing the effect of lateral position intubation on bronchial blocker placement during unilateral video-assisted thoracic surgery. Trials. 2025;26 (1): 553. doi: 10.1186/s13063-025-09287-7.

[3] Chen ZY, Lin YM, Wu JH, Fu YY, Xu XT, Li Y, et al. Does the periportal end of a double-lumen endobronchial tube need to be fixed to prevent dislocation of the cuffed end caused by a change in position? A randomized controlled trial. Ann Med. 2023;55(2): 2247422. doi: 10.1080/07853890.2023.2247422.

*Q3: Given the methodological limitations and the absence of demonstrated clinical benefit, the conclusion that the double-cuff bronchial blocker represents a meaningful alternative for airway management is not sufficiently supported by the current data.*

Response: Thank you for your important and rigorous comment on our conclusion. We fully respect your professional opinion and have revised the conclusion to be more conservative, evidence-based, and clinically precise.

We acknowledge that the study has some methodological limitations, including unfeasible operator blinding and a moderate sample size. However, we would like to clarify that our study did demonstrate a clear and significant clinical benefit: the DcBB group showed a markedly lower malposition rate (7.5% vs. 30%, P = 0.02) compared with the ScBB group, which is the key clinical issue during one-lung ventilation in thoracoscopic lobectomy. Reducing malposition can effectively decrease intraoperative interruptions, repeated bronchoscopic adjustments, and potential ventilation instability, which represents a meaningful clinical advantage.

In line with your suggestion, we have toned down the conclusion in the revised manuscript. We have moderated our conclusion and no longer claim that the DcBB is a “definitive” solution, but rather present it as a “promising option” for airway management.

We believe this revised conclusion is more objective and consistent with the actual findings of the study. We greatly appreciate your suggestion, which has helped us improve the scientific rigor and appropriateness of our manuscript.

Changes (page 3, line 45-48 and page 16, line 286-287): “and therefore may serve as a promising optional device for airway management…”
